# The contribution of PrEP programmes to triple elimination efforts: a cross-sectional study of status and opportunities

**DOI:** 10.3389/frph.2025.1637573

**Published:** 2025-09-18

**Authors:** Catherine E. Martin, Hlologelo Ramatsoma, Nthabiseng Koloane, Maletsatsi Monametsi, Sean Arries, Melanie Pleaner, Saiqa Mullick

**Affiliations:** Wits RHI, University of the Witwatersrand, Johannesburg, South Africa

**Keywords:** HIV, PrEP, syphilis, hepatitis B, triple elimination, prevention, contraception

## Abstract

**Background:**

The Triple Elimination initiative is a global effort aimed at eliminating vertical transmission of HIV, hepatitis B and syphilis. This paper describes HIV, syphilis and hepatitis B testing and diagnosis in young women and men accessing sexual and reproductive health services and identifies opportunities to integrate prevention interventions.

**Methods:**

The study was conducted in eight primary healthcare and four mobile clinics in South Africa, integrating HIV PrEP within prevention services. Programme data were collected and analysed from women and men ≥15 years accessing services for the first time between June 2023 and March 2024.

**Results:**

Of 10,007 clients, 89.4% were female and 65.5% 18─24 years. Overall, 70.9% were provided HIV PrEP. Among females, 16.8% were provided contraceptives for the first time. HIV was identified in 1.2% of males and 2.2% of females tested and with results available, syphilis in 5.6% and 5.0%, and hepatitis B in 1.7% and 0.9% respectively. An HIV diagnosis was less likely among older age groups and those enrolled in school and more likely among those with part-time relative to full-time employment. Syphilis was less likely among older age groups and those reporting consistent condom use. Hepatitis B was more likely among those who had used oral PrEP before.

**Conclusions:**

Opportunities for integrated prevention interventions, aligned to triple elimination, include condom programming, contraception, point-of-care testing, PrEP and vaccination. Integrated care delivered through HIV prevention programmes provides an opportunity to treat and prevent HIV, syphilis and hepatitis B, and offer contraception to prevent unintended pregnancies.

## 1 Introduction

Together HIV, viral hepatitis and sexually transmitted infections (STIs) account for 2.5 million deaths and 1.2 million new cases of cancer each year (1). Despite goals to eliminate viral hepatitis in sub-Saharan Africa by 2030, hepatitis-related deaths have increased from 1.1 million in 2019 to 1.3 million in 2022, and the estimated number of deaths due to hepatitis B now exceeds those of HIV (1). The unprecedented global increases in new adult and congenital syphilis cases since the COVID-19 pandemic have raised much alarm, requiring urgent interventions to reverse these trends (1, 2). Despite key achievements and declining HIV-incidence and HIV related deaths, new HIV infections remain disproportionately high in young women in sub-Saharan Africa and require accelerated efforts to achieve targets by 2025 (1). Africa has the highest burden in the world of HIV, syphilis and hepatitis B, yet has fallen behind other regions in reducing vertical transmission of these infections (3). In the African region there were an estimated 172,000 hepatitis B (0.4% of live births) (4) and 110,400 HIV vertical transmission events in 2022 (5), and an estimated 404,000 cases of congenital syphilis in 2016 (6). A global, integrated focus on “Triple Elimination” (7) has been initiated to address these three diseases, given their overlapping burden and modes of transmission, their synergistic effects which increase vertical transmission and adverse birth outcomes, and the similarity and availability of rapid diagnostic tests and effective prevention and treatment interventions (3, 7).

Much focus has been placed on integrating triple elimination interventions through existing maternal and child health (MCH) service delivery platforms, yet opportunities exist for triple elimination interventions beyond MCH settings. Substantial investment has been made in scaling access to oral pre-exposure prophylaxis (PrEP) for the prevention of HIV, particularly for young women. In South Africa, HIV PrEP is available at over 4,000 health facilities, and the country has initiated almost 1.5 million people on oral PrEP as of May 2024 (8). Given the common modes of transmission, HIV prevention services may offer an entry point to integrate key primary prevention interventions for HIV, syphilis and hepatitis B virus (HBV), thereby accelerating primary prevention efforts for all three diseases alongside improved access to screening, diagnosis and management.

The objectives of this paper are to describe the testing and diagnosis of HIV, syphilis and hepatitis B in a population of young women and men accessing sexual and reproductive health (SRH) services in eight primary healthcare clinics and four linked mobile clinics in South Africa; and to identify and discuss opportunities for integration of triple elimination efforts with a focus on HIV prevention and SRH programmes.

## 2 Methods

### 2.1 Study setting

This cross-sectional study is embedded within a large, ongoing Unitaid-funded implementation study (Project PrEP), which is evaluating the introduction of PrEP (including new PrEP methods) in primary healthcare settings through integrated HIV prevention and SRH services, with a focus on girls and young women 15─24 years. The project is implemented in eight fixed Department of Health facilities and four linked mobile clinics in four areas of South Africa: Tshwane (peri-urban area) in Gauteng, Gqeberha (peri-urban area) and Mthatha (rural area) in the Eastern Cape, and eThekwini (urban area) in Kwa Zulu Natal. These sites have been supported by Project PrEP to provide integrated, youth-friendly SRH services since 2018. Sites were selected to participate in Project PrEP based on the incidence of HIV and teenage pregnancy in their surrounding communities, as well as their proximity to learning institutions which serve young people. The first phase of the project supported the introduction of oral PrEP in South Africa and has been described elsewhere (9). The second phase is ongoing and has introduced the new PrEP methods dapivirine vaginal ring (DVR), since August 2023, and injectable cabotegravir (CAB-LA) since April 2024. Project service points within participating sites provide integrated HIV prevention and SRH services according to local guidelines, including HIV testing, PrEP, contraception, STI screening and management, with screening and linkage to gender-based-violence and mental health services provided. Clients known to be living with HIV and taking antiretroviral therapy are generally seen at other service points in the clinic, as are clients who are known to be pregnant, who are seen at service points providing antenatal care.

### 2.2 Data collection and analysis

This study utilized routine programme monitoring and evaluation data from women and men ≥15 years accessing SRH and HIV prevention services through Project PrEP, at their point of entry to project services. Data encompassed client demographics and socio-behavioural characteristics, documented in clinic files by the consulting healthcare provider. Data on pregnancy, contraception use, presence of STI signs and symptoms and STI treatment in the preceding three months were also documented. Additionally, data on the provision and results of rapid point-of-care HIV testing, pregnancy screening, syphilis testing, HBV testing, the initiation of PrEP and the provision of contraception were recorded. HIV testing was provided to consenting clients, following a two-step serial testing algorithm aligned with national HIV Testing Service guidelines (10), with two positive rapid test results confirming an HIV positive status, and a single negative rapid test confirming an HIV negative status. Pregnancy testing was conducted using a point-of-care (POC) urine human chorionic gonadotropin (hCG) test. In line with national PrEP guidelines (11), clients initiated on oral PrEP underwent baseline laboratory testing for hepatitis B surface antigen (HBsAg) and those pregnant or ≥30 years had their renal function assessed through a baseline serum creatinine and estimated glomerular filtration rate at the time of PrEP initiation. Furthermore, clients seeking services who, based on the clinician's assessment, had symptoms of syphilis, may have been exposed to syphilis, or were from a vulnerable population group (i.e., men who have sex with men, sex workers and their clients, adolescent girls and young women accessing SRH services, pregnant women, mobile workers) underwent syphilis screening using a Rapid Plasma Reagin (RPR) test conducted at local laboratories. Results from laboratory-based testing for Hepatitis B and syphilis were generally available within three days, through an online laboratory results system that could be accessed by the attending clinician. Clients received their laboratory results at their next visit or were recalled to the clinic for treatment or clinical care by the attending clinician if required. Clients with STI symptoms were treated on the same day according to a syndromic management approach (12), and asymptomatic clients on the day they returned for their results if positive for syphilis. Clients testing positive for HIV were initiated on antiretroviral treatment (ART) by the attending nurse, or referred for initiation within the same clinic, on the day of testing. Clients testing positive for HBsAg were referred for further evaluation and management by the doctor at the clinic.

We conducted a cross-sectional, descriptive analysis of routinely collected data from clients accessing clinical services for the first time at project sites between June 2023 and March 2024. To examine the association between independent variables and the binary dependent variables HIV, syphilis, and HBV test results, while holding other variables constant, we fit multivariable firth's logistic regression models. Given the pronounced imbalance in HIV status (195 HIV-positive vs. 9,219 HIV-negative), syphilis status (168 positive vs. 3,136 negative), and hepatitis B status (32 positive vs. 3,218 negative), Firth's penalized logistic regression was used to correct for bias in maximum likelihood estimates that can arise under such conditions and prevent overfitting (13). Statistical significance was assessed at a *p*-value cutoff of 0.05. Data were analysed using Stata statistical software, version 18.0 (14).

## 3 Results

Table 1 outlines the characteristics of the study population at their first visit, by biological sex. Of the 10,007 clients, 10.6% (*n* = 1,064) were male and 89.4% (*n* = 8,943) female. Most (65.5%, *n* = 6,556) were aged 18–24 years, with a higher proportion of females 15–17 years (16.4%, *n* = 1,463) compared to males (10.0%, *n* = 106), and a higher proportion of males ≥25 years (33.2%, *n* = 353) compared to females (17.1%, *n* = 1,529). A higher proportion of females (66.6%, *n* = 5,958) than males (46.8%, *n* = 498) were attending school or an educational institute. Most participants had completed secondary or a higher level of education, with a higher proportion of males (14.3%, *n* = 152) having completed tertiary education than females (8.3%, *n* = 743). Only 10.3% (*n* = 1,026) of the participants were employed full-time, higher amongst males (22.6%, *n* = 240) than females (8.8%, *n* = 786). Three-quarters of the participants were married or in a relationship (74.6%, *n* = 7,409). A higher proportion of males (22.3%, *n* = 202) compared to females (11.3%, *n* = 886) reported having sex under the influence of alcohol or drugs. Consistent condom use was reported for every sexual encounter by 15.9% (*n* = 1,349) of participants, similar among both males and females. Among females, a third (30.3%, *n* = 3,035) were using injectable contraceptives, 8.7% (*n* = 780) a subdermal contraceptive implant, 4.8% (*n* = 427) oral contraceptive pills and 0.7% (*n* = 68) an intrauterine contraceptive device (IUCD) on presentation to project sites. Overall, 1,506 (16.8%) females were provided contraceptives for the first time at their visit. There were 415 (5.1%) females who were pregnant or confirmed to be pregnant at their visit. STI signs or symptoms were documented for approximately a tenth (9.0%, *n* = 898) of participants, with 2.4% (*n* = 230) reporting being treated for an STI in the preceding three months. Similar proportions of males (9.5%, *n* = 98) and females (9.1%, *n* = 757) tested for HIV for the first time, whereas more females (14.4%, *n* = 1,203) than males (10.4%, *n* = 100) had used oral PrEP before. PrEP provision was approximately equal among males and females, at 70.9% (*n* = 7,019) overall.

**Table 1:** Characteristics of clients accessing SRH and HIV prevention services, by sex.

Variables	Male*	Female	Total
	(*N* = 1,064; 10.6%)	(*N* = 8,943; 89.4%)	(*N* = 10,007)
Age			
Median (Interquartile range)	22 (20, 28)	21 (18, 23)	21 (18, 24)
Minimum age, maximum age	15, 69	15, 68	15, 69
Age group			
15─17 years	106 (10.0%)	1,463 (16.4%)	1,569 (15.7%)
18─24 years	605 (56.9%)	5,951 (66.5%)	6,556 (65.5%)
25+ years	353 (33.2%)	1,529 (17.1%)	1,882 (18.8%)
Currently attending school or educational institute	498 (46.8%)	5,958 (66.6%)	6,456 (64.5%)
Highest educational level completed			
Less than primary	3 (0.3%)	24 (0.3%)	27 (0.3%)
Primary	273 (25.7%)	2,330 (26.1%)	2,603 (26.0%)
Secondary	636 (59.8%)	5,845 (65.4%)	6,481 (64.8%)
Tertiary	152 (14.3%)	743 (8.3%)	895 (8.9%)
Missing	0	1	1
Employment status			
Employed (full-time)	240 (22.6%)	786 (8.8%)	1,026 (10.3%)
Employed (part-time)	42 (3.9%)	298 (3.3%)	340 (3.4%)
Self-employed	58 (5.5%)	103 (1.2%)	161 (1.6%)
Unemployed	724 (68.0%)	7,753 (86.7%)	8,477 (84.7%)
Missing	0	3	3
Relationship status			
Single	302 (29.0%)	2,215 (24.9%)	2,517 (25.4%)
Married or in a relationship	741 (71.0%)	6,688 (75.1%)	7,409 (74.6%)
Missing	21	60	81
Had sex under the influence of alcohol or drugs^a^	202 (22.3%)	886 (11.3%)	1,088 (12.5%)
Used condoms every time they had sex^a^^,^^b^	127 (14.0%)	1,222 (16.1%)	1,349 (15.9%)
Reported or tested positive for pregnancy	NA	415 (5.1%)	
Used contraception before	NA	5,541 (62.0%)	
Current contraception used	NA		
None		3,999 (44.7%)	
Condoms only		627 (7.0%)	
Injectable		3,035 (33.9%)	
Implant		780 (8.7%)	
IUCD		68 (0.8%)	
Emergency contraceptive/Morning after pill		7 (0.1%)	
Oral contraceptive pill		427 (4.8%)	
Contraception provided for the first time	NA	1,506 (16.8%)	
STI signs and symptoms	109 (10.2%)	789 (8.8%)	898 (9.0%)
Treated for an STI in the last 3 months	21 (2.2%)	209 (2.5%)	230 (2.4%)
Used oral PrEP before	100 (10.4%)	1,203 (14.4%)	1,303 (14.0%)
First-time testing for HIV	98 (9.5%)	757 (9.1%)	855 (9.1%)
Prescribed PrEP	750 (70.5%)	6,341 (70.9%)	7,091 (70.9%)

* Includes five transgender females.

^a^ In the last month.

^b^ Among those who reported having sex (*n* = 7,254).

Table 2 presents data on the testing and diagnosis of HIV, hepatitis B, and syphilis at first visit, by sex and age. Among male clients, 96.8% (1,030/1,064) were tested for HIV, with 1.2% (*n* = 12) testing positive. Of the 693 (65.1%) males who were tested for syphilis, an active infection was confirmed in 5.6% (21/371) of those with results available, highest at 7.7% (8/104) among those ≥25 years. Of the 694 (65.2%) males screened for HBV, HBsAg was positive in 6/349 (1.7%) of those with results available, of whom two were co-infected with both syphilis and HBV. Among females, 93.7% (8,384/8,943) were tested for HIV, with 2.2% (*n* = 183) testing positive. Of the 5,634 (63.0%) females who were tested for syphilis, an active infection was confirmed in 5.0% (147/2,932) of those with results available, highest at 6.6% (33/499) among those 15–17 years. Of the 5,731 (64.1%) females screened for HBV, HBsAg was positive in 26/2,901 (0.9%) of those with results available, of whom five were co-infected with syphilis and HBV. Among clients diagnosed with hepatitis B, 15/32 (46.8%) were born prior to childhood HBV vaccine roll out in South Africa and 17/32 (53.1%) after.

**Table 2:** HIV, hepatitis B and syphilis testing among clients accessing SRH and HIV prevention services, by sex and age.

Variables	Males			
	15–17 years	18–24 years	25 + years	Total
	(*n* = 106; 10.0%)	(*n* = 605; 56.9%)	(*n* = 353; 33.2%)	(*N* = 1,064)
Tested for HIV	101 (95.3%)	577 (95.4%)	352 (99.7%)	1,030 (96.8%)
HIV positive	1 (1.0%)	8 (1.4%)	3 (0.9%)	12 (1.2%)
Tested for syphilis	66 (66.3%)	364 (60.2%)	263 (74.5%)	693 (65.1%)
Syphilis results available	41 (62.1%)	226 (62.1%)	104 (39.5%)	371 (34.9%)
Syphilis positive	3 (7.3%)	10 (4.4%)	8 (7.7%)	21 (5.6%)
Tested for Hepatitis B	67 (63.2%)	365 (60.3%)	262 (74.2%)	694 (65.2%)
Hepatitis B results available	38 (56.7%)	209 (57.3%)	102 (38.9%)	349 (50.3%)
Hepatitis B positive	1 (2.6%)	0 (0.0%)	5 (4.9%)	6 (1.7%)
Co-infections				
Syphilis and Hepatitis B^a^	1 (1.3%)	0 (0.0%)	1 (0.5%)	2 (0.3%)
Variables	Females			
	15–17 Years	18–24 Years	25+ Years	Total
	(*n* = 1,463; 16.4%)	(*n* = 5,951; 66.5%)	(*n* = 1,529; 17.1%)	(*N* = 8,943)
Tested for HIV	1,271 (86.9%)	5,649 (94.9%)	1,464 (95.7%)	8,384 (93.7%)
HIV positive	33 (2.6%)	126 (2.2%)	24 (1.6%)	183 (2.2%)
Tested for syphilis	920 (62.9%)	3,709 (62.3%)	1,005 (65.7%)	5,634 (63.0%)
Syphilis results available	499 (54.2%)	1,975 (53.2%)	458 (45.6%)	2,932 (52.0%)
Syphilis positive	33 (6.6%)	98 (5.0%)	16 (3.5%)	147 (5.0%)
Tested for Hepatitis B	927 (63.4%)	3,774 (63.4%)	1,030 (67.4%)	5,731 (64.1%)
Hepatitis B results available	498 (53.7%)	1,954 (51.8%)	449 (43.6%)	2,901 (50.6%)
Hepatitis B positive	2 (0.4%)	16 (0.8%)	8 (1.8%)	26 (0.9%)
Co-infections				
Syphilis and Hepatitis B^a^	0 (0.0%)	5 (0.1%)	0 (0.0%)	5 (0.1%)

^a^ Among those who had syphilis and Hepatitis B results available.

In the multivariable regression analysis (Table 3), participants aged 18–24 [adjusted odds ratio (aOR) = 0.47; 95% CI: 0.28–0.78] and those ≥25 years (aOR = 0.30; 95% CI: 0.15–0.61) had 53% and 70% lower odds of an HIV positive diagnosis, respectively, compared to those aged 15–17 years. Being currently enrolled in school or an educational institution was associated with 60% lower odds of an HIV positive diagnosis (aOR = 0.40; 95% CI: 0.26–0.62) relative to non-students and participants employed part-time had more than four times the odds of an HIV positive diagnosis compared with full-time employees (aOR = 4.46; 95% CI: 1.75–11.37). Syphilis diagnosis was associated with younger age and was less likely among consistent condom users (Table 3). Those aged 18–24 years had 38% lower odds of a syphilis diagnosis (aOR = 0.62; 95% CI: 0.39–0.99) and those ≥25 years had 57% lower odds (aOR = 0.43; 95% CI: 0.22–0.82), compared to adolescents aged 15–17 years. Participants who reported using condoms at every sexual encounter were 45% less likely to have a syphilis diagnosis than those who did not (aOR = 0.55; 95% CI: 0.32–0.96). Regarding hepatitis B diagnosis, those who had used oral PrEP before faced 2.5-times higher odds of hepatitis B than PrEP-naïve participants (aOR = 2.52; 95% CI: 1.04–6.11).

**Table 3:** Multivariable firth's logistic regression models of factors associated with HIV, syphilis, or hepatitis B positive test results.

Variables	HIV positive		Syphilis positive		Hepatitis B positive	
	Adjusted odds ratio (95% CI)	*p*-value	Adjusted odds ratio (95% CI)	*p*-value	Adjusted odds ratio (95% CI)	*p*-value
Sex						
Male	Reference		Reference		Reference	
Female	1.99 (0.89–4.45)	0.095	0.90 (0.55–1.47)	0.673	0.68 (0.25–1.81)	0.438
Age group						
15–17 years	Reference		Reference		Reference	
18–24 years	0.47 (0.28–0.78)	0.004	0.62 (0.39–0.99)	0.044	0.67 (0.20–2.28)	0.524
25+ years	0.30 (0.15–0.61)	0.001	0.43 (0.22–0.82)	0.011	1.84 (0.47–7.28)	0.383
Currently attending school or an educational institute						
No	Reference		Reference		Reference	
Yes	0.40 (0.26–0.62)	<0.001	0.70 (0.47–1.04)	0.075	0.56 (0.23–1.35)	0.196
Employment status						
Employed (full-time)	Reference		Reference		Reference	
Employed (part-time)	4.46 (1.75–11.37)	0.002	1.51 (0.63–3.62)	0.353	0.88 (0.14–5.64)	0.896
Self employed	2.29 (0.54–9.72)	0.262	1.88 (0.68–5.21)	0.222	1.78 (0.28–11.31)	0.542
Unemployed	2.04 (0.92–4.54)	0.080	0.89 (0.49–1.64)	0.715	1.14 (0.38–3.41)	0.816
Relationship status						
Single	Reference		Reference		Reference	
Married or in a relationship	0.94 (0.60–1.46)	0.771	0.81 (0.54–1.20)	0.288	0.92 (0.36–2.36)	0.858
Had sex under the influence of alcohol or drugs^a^						
No	Reference		Reference		Reference	
Yes	1.10 (0.64–1.89)	0.741	1.43 (0.95–2.15)	0.090	1.10 (0.43–2.83)	0.837
Used condoms every time they had sex^a^^,^^b^						
No	Reference		Reference		Reference	
Yes	1.45 (0.91–2.30)	0.114	0.55 (0.32–0.96)	0.035	0.82 (0.27–2.53)	0.730
Treated for an STI in the last 3 months						
No	Reference		Reference		Reference	
Yes	1.48 (0.57–3.88)	0.421	1.62 (0.60–4.37)	0.337	2.32 (0.43–12.60)	0.328
STI signs and symptoms						
Not present	Reference		Reference		Reference	
Present	1.23 (0.69–2.20)	0.480	1.05 (0.59–1.87)	0.875	2.04 (0.73–5.70)	0.172
Used oral PrEP before						
No	Reference		Reference		Reference	
Yes	0.91 (0.54–1.54)	0.721	1.04 (0.61–1.76)	0.894	2.52 (1.04–6.11)	0.040

^a^ In the last month.

^b^ Among those who reported having sex.

## 4 Discussion

In this large implementation study, focusing on integrated HIV prevention service delivery in primary care settings in South Africa, we identified HIV in 2%, syphilis in 5% and HBV in 1% of clients tested when accessing SRH services. The likelihood of testing positive for HIV and syphilis decreased with age, highlighting the importance of accessible prevention interventions for adolescents. In keeping with prior studies reporting the protective effect of school completion on HIV acquisition (15, 16), clients in this study who were attending a school or educational institute were less likely to be diagnosed with HIV than those who were not, whilst those who were employed part-time were more likely to be diagnosed with HIV than those employed full-time. Although condom use was not associated with HIV or hepatitis B in this study, those who consistently used condoms were less likely to test positive for syphilis than those who did not, underscoring the importance of condoms in the prevention of STIs. Prior use of oral PrEP was associated with testing positive for hepatitis B, possibly due to clients with known vulnerabilities and exposures self-identifying their need for prevention interventions. Almost all clients were tested for HIV at their first engagement with project services, including 9% of clients who tested for HIV for the first time. In addition, 17% of women were provided with contraception for the first time. Our results highlight the following opportunities for integration of triple elimination efforts within integrated SRH and HIV prevention programmes, focusing on the first pillar of triple elimination which looks at primary prevention interventions.

### 4.1 Condoms

Condoms are the only currently available multipurpose prevention technology, preventing pregnancy, HIV and other STIs; they are cost effective and should be the cornerstone of any integrated prevention package (17). However, consistent use of condoms remains a challenge and was observed to be low in this population, not dissimilar to reports from other PrEP programmes (18, 19). Low use of condoms among young women has been reported to be influenced by difficulty in negotiating use, fear of being perceived as unfaithful, and wanting to please partners (20, 21); with men reporting that forgetfulness and condoms being a barrier to pleasure impede consistent use (22). Condom use has been reported to increase among young women exposed to PrEP (18, 23), highlighting the benefits that may be achieved through strengthened integration, ongoing non-judgemental counselling, and incorporation of innovative approaches such as pleasure based messaging (17).

### 4.2 Contraception

Among women presenting for SRH services, opportunity exists to conduct pregnancy screening and provide contraceptive services. As highlighted through our study, almost half of women accessing services were not using any contraceptive at their first visit, but 17% were provided with contraception for the first time through integrated services. South Africa has a high unmet need for contraception, reported at 19% among women of reproductive age in a 2016 Demographic Health Survey (24); higher at 31% among adolescent girls and women 15─19 years and 28% among those 20─24 years of age (24, 25). There is also evidence of unintended pregnancy being high, especially in girls and young women. As shown in an analysis of the South African National HIV Prevalence, Incidence and Behaviour Survey, only a third of women reporting pregnancy in the last five years had desired the pregnancy; even lower at 10.1% among adolescent girls and women 15─19 years and 20.9% among those 20─24 years of age. Contraception use was low, particularly in younger women, with only 12.8% of those 15─19 years and 19.7% of those 20─24 years reporting using contraception (26, 27). In addition to well-known benefits relating to improved maternal and child health, contraception has been highlighted as a key, cost-effective intervention to prevent new infant HIV transmissions, even in the context of increased antiretroviral therapy (ART) coverage (28, 29). The risk of acquiring HIV during pregnancy and the postpartum period more than doubles as compared to non-pregnant women (30). A high prevalence of STIs and a high incidence of HIV have been reported among women seeking contraceptive services (31), highlighting the need and potential to address multiple health needs through integrated service provision. This project has previously reported the increased access to contraceptives that is achieved when integrated with HIV prevention services (32).

### 4.3 Point of care screening and testing

POC testing for STIs facilitates earlier diagnosis and linkage to treatment, and is acceptable and feasible in low and middle income settings (33). However, the availability and affordability of quality-assured point of care tests remain a barrier to access. The WHO recommends supply-side interventions, in conjunction with national policy updates and funding, to improve early detection and treatment, resulting in greater impact in ending the epidemics and promoting health coverage (34). Our study highlights the benefits of POC screening and testing, with almost all clients who accessed services tested for HIV, and 9% of clients testing for HIV for the first time. The high burden of syphilis identified in this study points to the impact that may be achieved through the integration of POC syphilis tests within SRH programmes. The limitations of laboratory-based testing, including the loss to follow-up observed in our analysis (indicated by the low proportion of available results), are notable. Although only currently implemented in antenatal care settings in South Africa, increased syphilis screening and treatment may be achieved with the integration of the currently available dual HIV/syphilis POC test within HIV prevention services. These tests are acceptable, feasible and more cost effective than single tests (35), although implementation data from the region, outside of antenatal care, is limited (36).

South African national guidelines recommend HBV surface antigen screening for all pregnant women during the first trimester (37) and for all clients initiating oral PrEP (11). However, this is currently conducted through laboratory-based testing, is not routinely implemented for pregnant women, and POC rapid tests are not currently available within the national health system in South Africa (38). Integrating HBV screening with HIV prevention programmes could facilitate progress towards HBV elimination efforts (39), and could be strengthened with POC testing. Scaling testing to those who may benefit has been identified as one of the greatest challenges in achieving elimination targets for viral hepatitis (40). Use of a POC HBV tests has been shown to be feasible in antenatal care settings in South Africa (41, 42). However, further implementation research on the integration of rapid POC HBV testing within HIV prevention programmes in our context may be required.

The use of self-testing may provide further opportunity to reach partners and social networks with screening and testing services, optimising identification, and early treatment of infection to prevent transmission and reinfection. HIV self-test kits are available in South Africa, and secondary distribution has been acceptable in antenatal care settings (43). Further opportunities for integration of syphilis self-testing, when available, also exist (44).

### 4.4 Pre-exposure prophylaxis

In keeping with other implementation studies in the region (45, 46), and as reported previously for this project (9), we observed a high uptake of HIV PrEP when offered within a package of integrated prevention services. PrEP has been shown to be highly effective in preventing HIV and is a key component of combination HIV prevention and Triple Elimination efforts (7, 47). Initiatives to accelerate access to meet global prevention targets are, however, still required (48). The introduction of newer, long-acting PrEP methods has the potential to further enhance PrEP uptake and effective use for the prevention of HIV. One advantage of oral PrEP over long-acting PrEP methods, however, is its activity against HBV. Tenofovir disoproxil fumarate (TDF) or tenofovir alafenamide (TAF) within the oral PrEP combination of TDF and emtricitabine (FTC), are potent inhibitors of HBV viral replication and effective at both preventing HBV acquisition and suppressing HBV replication (39, 49). Globally, challenges with testing and treatment for chronic HBV remain, with less than 3% of people living with chronic hepatitis B globally treated in 2022 (50). Oral PrEP services thus provide an opportunity to facilitate integrated prevention interventions for both HIV and HBV, whilst improving access to treatment for people with HBV (39, 49). It has however been recommended, due to concerns about hepatitis reactivation, or “flares”, in people with chronic HBV who discontinue TDF/FTC, that there is closer follow up and monitoring of those with HBV infection who initiate TDF/FTC PrEP (49). The lack of benefit against HBV with long-acting PrEP methods could be overcome through integrated HBV screening regardless of PrEP method and ensuring availability of a choice of PrEP methods to suit different individual needs (49).

### 4.5 HBV vaccination

HBV vaccination has been a key success in combatting hepatitis infection, with widespread adoption of childhood immunization globally (40). In South Africa, substantial declines in hepatitis B incidence have been achieved since the inclusion of HBV vaccination for children as part of the expanded programme for immunization (EPI) since 1995 (51, 52). Whilst most programming is focused on childhood vaccination, and intensified focus and strengthening of birth dose vaccination more recently (40), there is opportunity to provide catch up vaccination to non-immune adults, especially those with HIV, who inject drugs or who may be exposed through sexual transmission, as is recommended in national guidelines (37). Within our study population, approximately half of the cases of hepatitis B identified were amongst clients born after the introduction of childhood immunization. Where HBV screening is integrated within HIV prevention programmes, additional laboratory testing would need to be conducted to confirm immunity (37), but the three dose HBV vaccination schedule can be aligned to PrEP follow up visits to facilitate integration (39).

### 4.6 Linkage to treatment

Linkage to care and treatment for individuals diagnosed with HIV, HBV, or syphilis through SRH programmes should be prioritized and is the second pillar of triple elimination efforts. Ideally this should be provided on the same day, by the same healthcare provider and at the same service point, to facilitate integrated care. National guidelines in South Africa recommend a first line antiretroviral therapy (ART) regimen which includes TDF and lamivudine (3TC), which has the benefit of treating both HIV and hepatitis B (39, 53).

### 4.7. Limitations

Although this study was conducted across four geographical areas of South Africa, the sampling frame did not include a representative sample of all SRH service users and the findings may not be representative of other contexts, particularly where the burden of HIV, HBV and syphilis differs. These data were collected from healthcare users who had presented for SRH and HIV prevention services and may not fully represent populations who have not accessed or experience barriers to accessing healthcare services. It is also notable that clients known to be living with HIV or who were pregnant would likely have received services at other service points in the clinic, and this data would not be representative of these populations. Incomplete laboratory results, particularly for syphilis and hepatitis B, may also have introduced bias. In addition, the lack of data on treatment outcomes for clients diagnosed with HIV, hepatitis B or syphilis and the cross-sectional study design limits analysis of outcomes and trends over time. The proposed opportunities for integration of triple elimination efforts that are discussed, are drawn from the data in our analysis and literature from the field but would be further supported by implementation research to determine their acceptability, feasibility, and cost effectiveness in specific contexts.

## 5 Conclusion

There are multiple opportunities for integrated prevention interventions, aligned to triple elimination efforts, including condom programming, contraception, POC testing, PrEP and vaccination. Leveraging investments and models of integrated care delivered through HIV prevention programmes focused on girls and young women may provide additional opportunity for triple elimination efforts by treating and preventing HIV, syphilis and hepatitis B prior to women becoming pregnant, in addition to offering integrated contraception services to prevent unintended pregnancies.

## Data Availability

The raw data supporting the conclusions of this article will be made available by the authors, without undue reservation.
